# Newborn Screening and Treatment of Phenylketonuria: Projected Health Outcomes and Cost-Effectiveness

**DOI:** 10.3390/children8050381

**Published:** 2021-05-12

**Authors:** Huey-Fen Chen, Angela M. Rose, Susan Waisbren, Ayesha Ahmad, Lisa A. Prosser

**Affiliations:** 1 Department of Health Management and Policy, School of Public Health, University of Michigan, Ann Arbor, MI 48109, USA; hfenchen@umich.edu; 2 Susan B. Meister Child Health Evaluation and Research Center, Department of Pediatrics, Medical School, University of Michigan, Ann Arbor, MI 48109, USA; angmrose@med.umich.edu; 3 Department of Pediatrics, Harvard Medical School, Boston, MA 02115, USA; Susan.Waisbren@childrens.harvard.edu; 4 Division of Pediatric Genetics, Metabolism and Genomic Medicine, Department of Pediatrics, Medical School, University of Michigan, Ann Arbor, MI 48109, USA; ayeshaah@med.umich.edu

**Keywords:** phenylketonuria, cost-effectiveness, sapropterin dihydrochloride, newborn screening

## Abstract

The objective of this study was to evaluate the cost-effectiveness of newborn screening and treatment for phenylketonuria (PKU) in the context of new data on adherence to recommended diet treatment and a newly available drug treatment (sapropterin dihydrochloride). A computer simulation model was developed to project outcomes for a hypothetical cohort of newborns with PKU. Four strategies were compared: (1) clinical identification (CI) with diet treatment; (2) newborn screening (NBS) with diet treatment; (3) CI with diet and medication (sapropterin dihydrochloride); and (4) NBS with diet and medication. Data sources included published literature, primary data, and expert opinion. From a societal perspective, newborn screening with diet treatment had an incremental cost-effectiveness ratio of $6400/QALY compared to clinical identification with diet treatment. Adding medication to NBS with diet treatment resulted in an incremental cost-effectiveness ratio of more than $16,000,000/QALY. Uncertainty analyses did not substantially alter the cost-effectiveness results. Newborn screening for PKU with diet treatment yields a cost-effectiveness ratio lower than many other recommended childhood prevention programs even if adherence is lower than previously assumed. Adding medication yields cost-effectiveness results unlikely to be considered favorable. Future research should consider conditions under which sapropterin dihydrochloride would be more economically attractive.

## 1. Introduction

Phenylketonuria (PKU, OMIM 212600) is an autosomal recessive inherited disorder of amino acid metabolism, affecting 5.3 to 7.4 newborns per 100,000 births in the US [1,2,3]. Mutations in the phenylalanine hydroxylase (*PAH*) gene prevent affected individuals with PKU from metabolizing the amino acid, phenylalanine [2]. If untreated, the excess phenylalanine can lead to severe intellectual disability and seizures [4]. Even slightly suboptimal phenylalanine levels can result in subtle neurocognitive deficits, such as slower information processing and memory impairments [2].

The primary treatment approach for PKU is dietary. Individuals with PKU are recommended to follow a strict low phenylalanine diet, including medical formula and specially formulated low-protein foods [5]. In 2007, sapropterin dihydrochloride (tetrahydrobiopterin (BH4)) was approved by the U.S. Food and Drug Administration for treatment of PKU [6]. This oral medication improves phenylalanine concentrations in a subset of individuals with PKU with BH4–responsive PKU, with 20–44% of individuals with PKU reported to be responsive in clinical trials [7,8,9].

While dietary treatment has demonstrated effectiveness in preventing intellectual disability, this requires strict adherence to the dietary recommendations [2]. Recent studies show that adherence to this restrictive diet is challenging and adherence rates may be lower than previously assumed [10,11,12]. Drug treatment with sapropterin dihydrochloride for eligible individuals with PKU may allow some individuals to liberalize their diet and therefore represents an important treatment option for PKU.

PKU was the first disorder screened for at a national level in the US [13] with the goal of early identification and treatment leading to improved outcomes for individuals with PKU. Previous economic evaluations have shown substantial cost savings associated with newborn screening for PKU and dietary treatment for diagnosed individuals [13,14,15,16,17,18]. However, these evaluations did not include variable adherence to the dietary treatment or the consideration of sapropterin dihydrochloride as an additional treatment option, which is highly costly (approximately $50,000–$120,000 per individuals per year) [19]. The objective of this study was to evaluate the cost-effectiveness of newborn screening and treatment for phenylketonuria (PKU) given new findings on adherence to dietary recommendations and to include the availability of sapropterin dihydrochloride as a treatment option concurrent with dietary treatment for select individuals with PKU.

## 2. Materials and Methods

A state–transition model was developed to evaluate the cost-effectiveness of newborn screening (NBS) and treatment of PKU. The target population was a hypothetical newborn cohort, and the model was designed to simulate lifetime cost and health outcomes of the cohort under different intervention strategies.

Four intervention strategies were compared in the model in which the cohort was either assumed to be diagnosed through newborn screening (NBS) or the clinical setting (clinical identification, CI), and assumed to receive diet treatment only or medication (sapropterin dihydrochloride) combined with diet treatment: (1) Clinical identification with diet treatment (CI/diet); (2) Newborn screening with diet treatment (NBS/diet); (3) Clinical identification with diet and medication (sapropterin dihydrochloride) (CI/diet with medication); and (4) Newborn screening with diet and medication (sapropterin dihydrochloride) (NBS/diet with medication).

Model inputs include parameters for epidemiology, costs, and health-related quality of life associated with PKU (Table 1, Table 2 and Table 3, Tables S1–S5). State transition probabilities, treatment effects, and adherence rates were derived from published literature and supplemented by expert opinion. Primary data from a national survey fielded to US adults for health-related quality of life, and a survey fielded to individuals with PKU (or their parents) informed inputs for treatment costs, time associated with recommended treatments, and quality of life adjustments [19,20].

### 2.1. Model Structure and Assumptions

**Figure 1:**
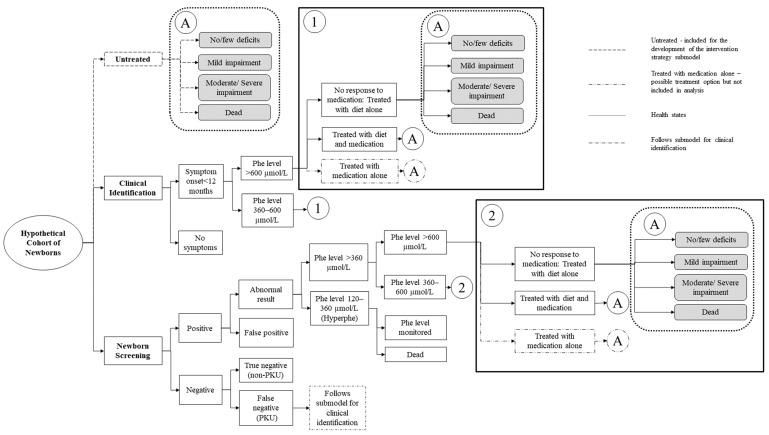
Model framework.

The model framework is shown in Figure 1 with three submodels to represent untreated newborns (natural history), newborns clinically identified, and newborns identified through newborn screening.

### 2.2. Epidemiology: Natural History of PKU

**Figure 2:**
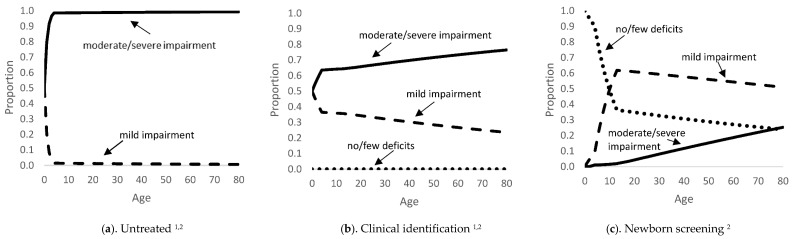
Proportion of Hypothetical Cohort of PKU Patients by Health State and Age–diet treatment only (no medication), partial adherence. ^1^ Individuals with PKU that are untreated or identified through clinical identification start with mild or moderate/severe impairment. ^2^ These figures only reflect the proportion of individuals alive at that age and does not include those that have died.

While it is unlikely for individuals with PKU today to be untreated, we included a submodel that represents the natural history of PKU (untreated) for the development of the intervention strategy submodels. The natural history submodel included four health states for individuals with PKU: no/few deficits, mild impairment, moderate/severe impairment, and dead (Figure 1). The health states are not defined by Phe level but are correlated with the level of impairment. Some simplifying assumptions were made: (1) once individuals have transitioned to a worse health state they cannot return to a better state, and (2) individuals must pass through intermediate health states to transition to a more severe state. We assume the health states of the individuals with PKU are evaluated every year in the model, i.e., model cycle length is one year. The lifetime trajectories through health states of these submodels are shown in Figure 2 (and Figure S1). Probability inputs were derived from published literature and supplemented by expert opinion [38,39].

### 2.3. Clinical Identification and Newborn Screening Submodels

In the clinical identification strategies (Figure 1), newborns were assumed to have symptom onset within a year and diagnosed in the clinic setting. Newborns diagnosed with PKU were then treated with diet treatment only or diet treatment combined with medication. In the newborn screening strategies, newborns were assumed to receive initial screening at birth followed by additional confirmatory testing for those who screen positive. They were assumed to receive the same treatment as those in the clinical identification strategies but with earlier initiation of treatment. We assumed equal size cohorts of individuals with PKU in both NBS and clinical identification submodels.

### 2.4. Treatment Interventions

Two treatment interventions were included: diet treatment and medication (sapropterin dihydrochloride). The treatment intervention effectiveness is incorporated into the model by reducing the probability of transition to a worse health state. In our model, we assume everyone who is eligible for sapropterin will utilize it in the medication strategies. In determining whether individuals with PKU received diet treatment only or diet treatment combined with medication, data from literature were used and only individuals with PKU who are responsive to medication will receive this option, e.g., for individuals with PKU with Phe level 360–600 μmol/L, the probability of responding to medication was 0.81 (range: 0.79 to 0.83), and therefore, it is assumed in base case that 81% of this group of individuals with PKU responded to medication and received both diet treatment and medication, while the 19% were unresponsive to medication and treated with diet treatment only [23]. For individuals with PKU with Phe level >600 μmol/L, the probability was assumed to be 0.315 (range: 0.07 to 0.6) [23].

For individuals with PKU that fully adhere to their treatment, medication was assumed to be 100% effective in reducing the probability of disease progression, and 99% for diet treatment, and these were assumed to be fixed over time [38,39,40]. The effectiveness of treatment is reflected in the model by applying a percent reduction to the transition probabilities for disease progression. For example, a treatment that is 99% effective will reduce the transition probability by 99%. When adherence is also incorporated into the model, the level of adherence is considered together with the treatment effectiveness in a combined function. In the case of partial adherence, the effectiveness of treatment would be reduced by the adherence rate, e.g., if treatment effectiveness is 99% and adherence is 50%, then treatment effectiveness with partial adherence would only reduce the transition probability for disease progression by 44.5%.

Individuals with PKU with Phe level between 120 and 360 umol/L (hyperphe) were assumed to be monitored rather than treated in the model to be consistent with current practice.

### 2.5. Adherence Rates

For base–case analysis, individuals with PKU were assumed to have partial adherence rates for diet treatment and medications. Partial adherence rates were derived from literature. For diet treatment, partial adherence rates were based on age and ranged from 37.5% to 88%, while medication adherence was assumed to be around 65.5% (Table 1). A scenario analysis was conducted to explore the results of assuming full diet treatment and medication adherence rate, as well as alternative rate for the age group of 18 years old and above.

### 2.6. Mortality Rates

US life tables were used to estimate the annual probability of dying (Table S5). Individuals with PKU were assumed to have the same mortality rate as a person without PKU [40].

### 2.7. Costs and Resource Use

Two costing perspectives were used: the healthcare sector perspective and the societal perspective. The healthcare sector perspective included direct medical costs, and the societal perspective further includes relevant non-health care costs (in this case, caregiver’s time cost and individuals with PKU’s special education costs).

Direct medical costs included newborn screening and follow-up confirmatory testing costs, laboratory and developmental testing costs, visit costs, food costs, and medication costs. Visit costs included direct medical costs of visiting dieticians, geneticists, metabolic specialists, primary care providers, and psychologists (developmental testing costs). Food costs included medical formula and low protein food costs. However, given the increasing availability of low protein food in recent years, it is debated whether low protein food costs should be included as direct medical costs. Therefore, low protein food costs were varied in the scenario analysis using the healthcare sector perspective to explore this difference.

Medication costs were assigned using average wholesale prices in base–case analysis. Prices were calculated based on drug (sapropterin dihydrochloride) cost per 100 mg tab, recommended initial dose and maintenance dose per kg from literature, and weight from the Anthropometric Reference Data for Children and Adults: United States 2007–2010 [3,20,21,22]. The drug pricing negotiated by the Department of Veterans Affairs (VA-negotiated drug pricing schedule) was used in scenario analysis [28].

Non–medical costs included caregiver’s time costs for visits and food preparation, and individuals with PKU’s special education costs. Time costs were estimated based on the hours from a survey [19] and wage cost per hour [34]. Detailed information is shown in Table S2.

For the hyperphe population, costs included newborn screening and follow-up confirmatory testing costs, and monitoring costs (laboratory tests, time and visit costs for metabolic specialists and primary care providers). All costs were in 2017 US dollars and were adjusted using the Gross Domestic Product (GDP) deflator.

### 2.8. Health-Related Quality of Life

The primary health outcome measure in the analysis was the quality-adjusted life year (QALY). To generate QALYs, each health state is assigned a health utility weight. Utility weights are multiplied by the duration of time spent in each health state and summed across years to calculate QALYs. Health utilities typically range from 1 for perfect health to 0 which is equivalent to being dead. The health utility weights for PKU health states were determined from a separate study that used time trade-off (TTO) questions fielded to a national community-based sample [20]. In our study, values for mild symptoms ranged by age from 0.64 to 0.81 while values for moderate/severe symptoms ranged from 0.56 to 0.68 (Table 3). In scenario analysis, we adopted utility values determined from the same survey fielded to a sample of individuals with PKU. A scenario analysis including caregiver disutilites was also conducted to explore the potential effect of family spillover effects (Table 3).

### 2.9. Analysis Plan

A societal perspective was used as the primary analytic perspective to capture non-health care costs (e.g., time costs associated with adhering to the prescribed dietary recommendations), which are significant for individuals with PKU. We also conducted an analysis using the healthcare sector perspective for comparison (scenario analysis). Primary outcomes included costs, quality-adjusted life years (QALYs), and incremental cost-effectiveness ratios (ICERs), where the ICER is the ratio of the difference in costs divided by the difference in quality-adjusted life years between strategies [41]. The impact inventory for this study is presented in Table S6.

The base–case analysis was conducted from the societal perspective where individuals with PKU were assumed to be only partially adherent to recommended diet treatment and medication. Both costs and quality-adjusted life years were discounted at 3% to adjust for the differential timing of the outcomes in the future [42]. The analysis was conducted using TreeAge Pro 2020 version R1 (TreeAge Software, Inc., Williamstown, MA, USA).

We conducted one-way sensitivity analysis for the parameters to test the robustness of the model within most likely ranges of the parameters. Additional scenario analyses were conducted: assuming individuals with PKU are fully adherent, adopting the experienced PKU sample utility, using the VA-negotiated drug pricing schedule, and including caregiver disutility.

## 3. Results

### 3.1. Base–Case Analysis

For a cohort of 1000 individuals diagnosed with PKU, the base–case analysis results showed that NBS strategies had higher costs and QALYs when compared to the CI strategies (Table 4). When compared with CI/diet, NBS/diet had incremental costs of $2139 and incremental QALYs of 0.334 QALYs, which resulted in an ICER of $6400/QALY. Costs increased significantly when sapropterin dihydrochloride was used concurrently with diet treatment. Using medication with NBS/diet resulted in an ICER of $16,135,000/QALY, with incremental costs of $65,532 and incremental QALYs of 0.004 QALYs. When compared with NBS/diet, CI/diet with medication yielded lower QALYs (0.333 QALYs lower) and higher costs when medication was added (the strategy is considered “dominated”) and is not considered a favorable strategy.

### 3.2. Sensitivity Analysis

#### One–Way Sensitivity Analysis

**Figure 3:**
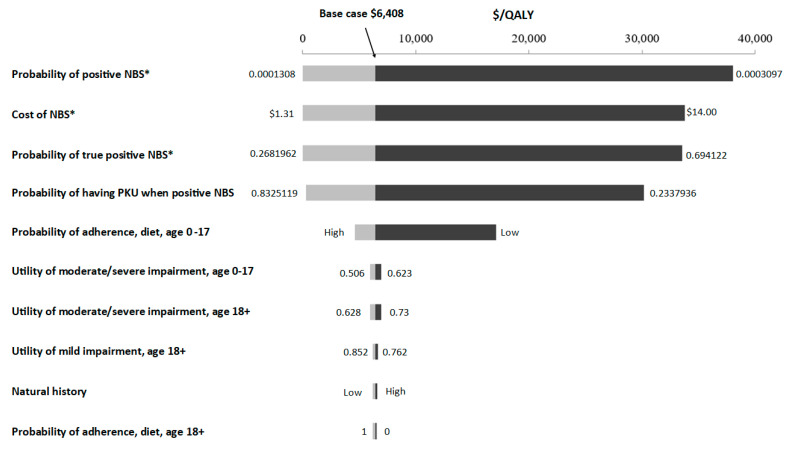
One–way sensitivity analysis for NBS/diet when compared with CI/diet, ICER. * For these variables, when the value decreased to a certain threshold, the reference case switched to NBS/diet and becomes a cost–saving strategy when compared to CI/diet the ICER is negative with NBS/diet as reference case, therefore, 0 is used here to indicate that NBS/diet is the more cost–effective strategy when the value is lower, i.e., when the cost of NBS decreases, when the probability of NBS screened positive decreases, and when the probability of true positive decreases, all these would turn NBS/diet to a more favorable strategy.

For the NBS/diet strategy, the probability of NBS screened positive had the most impact, the second most important variable was the cost of newborn screening, and the third was the probability of a confirmed true positive given a positive initial screen when varying input values across a plausible range (Figure 3). For NBS/diet with medication, key variables were treatment adherence rates and health state utilities (Figure S2). Additional information is shown in Table S7.

### 3.3. Scenario Analysis

#### 3.3.1. Healthcare Sector Perspective

Using a healthcare sector perspective, the rankings of the cost-effectiveness of the four strategies remained the same (Table S8). Overall costs decreased when using the healthcare sector perspective. The ICER for NBS/diet, when compared to CI/diet, increased to $15,339/QALY (versus societal perspective: $6408/QALY). CI/diet with medication remained dominated by NBS/diet, while the ICER for NBS/diet with medication when compared with CI/diet with medication was similar: $16,135,836/QALY (versus societal perspective: $16,135,442/QALY).

#### 3.3.2. Full Adherence to Dietary Treatment and Medication

The base case analysis assumed partial adherence reflecting recent data. In a scenario analysis assuming all individuals were fully adherent, all intervention strategies yielded higher costs and higher QALYs (Table S9). The ICER decreased to $4452/QALY for NBS/diet when compared to CI/diet, while NBS/diet with medication strategy remained greater than $15 million/QALY and CI/diet with medication remained an unfavorable strategy.

We further explored the scenario that many individuals may go through a period of poor metabolic control during adolescence but then return to better control in their twenties. We modeled this scenario by increasing the adherence rate of the individuals in the age group of age 18 and above (Table S9). Compared to the base–case assumption of 37.5% adherence rate for ages 18 and above, assuming adherence increased to rates from 50% to 100% following adolescence resulted in slightly lower ICERs but did not change the ranking of strategies. In this scenario analysis, it is assumed, aligned with base–case assumptions, that individuals are not able to move to a better health state but will have lower rates of worsening health associated with higher adherence.

#### 3.3.3. Using Experienced Individuals with PKU Ratings for Quality of Life Adjustments

When the utility weights from an experienced PKU sample were used instead of community weights, the ICER increased to $7380/QALY for NBS/diet compared to CI/diet, and NBS/diet with medication strategy increased significantly to >$25,000,000/QALY (Table S10).

#### 3.3.4. Using the VA-Negotiated Drug Pricing Schedule

When the VA-negotiated drug pricing schedule cost were used, the ICER decreased by 19% to $13,049,437/QALY for NBS/diet with medication (Table S11).

#### 3.3.5. Including Caregiver Disutility

When caregiver disutility was included, the ICER decreased to $4990/QALY for NBS/diet compared to CI/diet, and NBS/diet with medication strategy decreased to $12,430,733/QALY (Table S12).

## 4. Discussion

In this study, we evaluated the projected cost-effectiveness of newborn screening and treatment for PKU incorporating new data on adherence rate for dietary treatment and a newly available medication (sapropterin dihydrochloride). From a societal perspective, we found that NBS/diet yielded an ICER of $6408 per QALY when compared to CI/diet. This is regarded to be a favorable cost-effectiveness ratio using a conventional thresholds of $100,000–$150,000 per QALY in the US [43]. Adding medication to dietary treatment increased the cost significantly, and yielded cost-effectiveness results >$16 m/QALY, a range that would be considered unfavorable even using adjusted standards that have been suggested for rare conditions, such as the willingness–to–pay thresholds ($50,000 per QALY to $500,000 per QALY) for ultra–rare diseases proposed by the Institute for Clinical and Economic Review (ICER). Although it should be noted that the population of individuals with PKU is slightly larger than the ICER definition for an ultra–rare disease [44].

In the US, previous studies on the economic evaluation of newborn screening for PKU have demonstrated the benefits of screening [13,14,18,45]. Three earlier studies performed during the 1970s to 1980s conducted cost–benefit analysis, which explored the costs and the benefits both in terms of monetary values. One study focused on estimating the avoided cost for institutionalized individuals with PKU if they were not detected through newborn screening and found that newborn screening was cost–saving with the far lower cost of detection and treatment [13]. The other two studies compared the cost of establishing program with the avoided cost of institutionalization and both concluded that PKU screening program is a beneficial program [18,45]. A 2006 cost–utility analysis study of seven independent newborn screening strategies, including newborn screening for PKU, projected that PKU screening is cost–saving for the society in the long–term [14].

In recent years, sapropterin dihydrochloride has become available to individuals with PKU, which provides the individuals the opportunity of having a more flexible diet while maintaining acceptable Phe levels. While previous economic analyses clearly show that newborn screening program for PKU is a beneficial policy, sapropterin dihydrochloride was not available when these earlier studies were conducted. This raises the question of whether it would still be a favorable policy when this high cost drug treatment is taken into account. Our study, to our best knowledge, is the first to address the economic impact of a long–term newborn screening policy for PKU in the context of this available medication and finds that adding sapropterin dihydrochloride to the recommended dietary treatment yields cost-effectiveness results that are unlikely to be considered favorable.

The high cost of sapropterin dihydrochloride has a significant negative impact on the favorability of intervention strategies that included medication in our analysis. Even when using the lower VA-negotiated drug pricing schedule, strategies that included medication as part of the treatment plan remained unfavorable. To understand scenarios in which medication strategies could be considered favorable assuming a willingness–to–pay threshold of $100,000 per QALY, we conducted a threshold analysis to identify conditions under which these strategies would be favorable. For NBS/diet with medication to be favorable, medication costs would need to be less than $309 per individual with PKU per year, depending on age. For both NBS/diet with medication and CI/diet with medication strategies to be favorable, the cost would need to be below $1000 per individual per year depending on age. This is substantially lower than the current pricing of sapropterin at approximately $50,000 to $120,000 per individuals per year [19].

Another difference from previous studies is that previous studies did not address the issue of adherence rate, which suggests that they assumed full adherence to the recommended treatment. Recent studies, however, have shown that the majority of individuals with PKU do not fully adhere to recommended treatments [24,25], which suggests assuming full adherence would potentially over–estimate the effectiveness of the treatment. Based on our results, assuming partial adherence did yield slightly lower effectiveness then when compared to full adherence. In our model, cost was also adjusted as a result of partial adherence, though some may argue that the costs for partial adherence may be the same given some individuals might pay for the treatment but not adhere to the treatment. While assuming partial adherence did result in slightly lower ICER values, the ranking of cost-effectiveness outcomes remains unchanged.

There is, however, one subgroup who could potentially benefit most from receiving medication treatment: the subgroup of individuals with PKU who have low adherence to recommended dietary treatment, but high adherence to medication treatment. An exploratory analysis was conducted to estimate results for this scenario in which NBS/diet with medication with full adherence was compared to CI/diet with partial adherence rate. For this subset of individuals with PKU, the ICER is projected to be $202,862 per QALY, which would not meet conventional thresholds, but would be considered favorable under an alternative willingness–to–pay threshold of $500,000 per QALY proposed by the Institute for Clinical land Economic Review (ICER) for treatments for ultra–rare diseases [44].

The societal perspective included cost from the healthcare sector perspective and non-medical costs such as time costs for visits and food preparation and special education costs. It is important to capture these additional costs as treatments for individuals with PKU begins at a young age and families play a significant role in providing care. Even for adults, there are substantial time costs associated with having PKU [19]. Results using the societal perspective that include these important time costs are more favorable compared to results from the healthcare sector perspective.

Our study has limitations, including that there are still very scarce data on long–term outcomes for individuals with PKU. The model was based on published data on PKU outcomes through age 15 [38,39]. Extrapolations into later years were based on extending the function based on these earlier time points and expert opinion. To compensate for this, clinical experts were included in the model development process to assure the model reflected experiences consistent with the clinical setting. In addition, due to the size of the modeled cohort and the complexity of the disease model, we did not conduct probabilistic sensitivity analysis, as it was not feasible due to computational limitations.

Another limitation is that this analysis assumes that all patients eligible for sapropterin will elect to utilize this treatment option; however, in practice uptake of this treatment option may be lower. In addition, we did not include possible mental health conditions such as anxiety or depression that may be associated with PKU [46,47]. Possible seizures associated with untreated PKU were also not included due to the scarcity of data availability.

Another limitation in this study is its focus only on benefits to affected individuals. Although not included in the models described here, therapy with sapropterin may improve metabolic control during pregnancy in mothers with PKU, which, in turn, may improve outcomes of offspring, specifically preventing serious congenital heart disease, low birth weight, microcephaly, and intellectual disabilities [48,49,50].

In addition, there have been studies that discussed the partial reversibility of IQ deficits in late–treated individuals with PKU [51,52]. In our base–case model, we assumed that the health states in our model are irreversible, therefore, we conducted a scenario analysis to explore the assumption where late–treated individuals with PKU have a probability of recovering to a better health state when receiving diet treatment at an early age. NBS/diet was compared with CI/diet and yield an ICER of $15,000/QALY, which is still much lower than conventional thresholds (Table S13).

Another limitation is that given that there may be additional non-health effects that are not captured in our study, which are not typically captured in QALYs, such as the increased opportunity for adults to engage in common life activities such as employment and marriage.

## 5. Conclusions

Contrary to earlier studies which demonstrated cost savings associated with newborn screening for PKU, our study reports a net investment required for health gains but with a cost-effectiveness ratio far lower than many other recommended childhood prevention programs. The addition of medication treatment with sapropterin dihydrochloride to the recommended dietary treatment yields cost-effectiveness results that are unlikely to be considered favorable. However, there are multiple variables that cannot be fully explored at present based on the scarcity of available data. Future research is needed to explore under what conditions the addition of sapropterin dihydrochloride to diet treatment could be economically attractive.

## Figures and Tables

**Table 1:** Epidemiology Inputs

	Base–Case	Range for Sensitivity Analysis	Reference
**Newborn screening**			
Probability false negative screen	0	––	[21,22]
Probability positive screen	0.0002064	0.0001308–0.0003097	[3]
Probability positive screen, confirmatory testing|positive initial screen	0.4782609	0.2681962–0.694122	[3]
Probability PKU|positive confirmatory test	0.5454545	0.2337936–0.8325119	[3]
**Probability Phe level 360–600|PKU**	0.3770197	0.3366195–0.418732	[23]
**Treatment** ^1^			
Probability of responding to medication|Phe level 360–600, NBS	0.81	0.79–0.83	[23]
Probability of responding to medication|Phe level > 600, NBS/CI	0.315	0.07–0.6	[23]
Treatment effect–Diet treatment	0.99	––	Assumption ^2^
Treatment effect–Medication	1	––	Assumption ^3^
**Adherence rate** ^1^			
Diet treatment			
Age 0 to 3	0.88	0–1	[24], assumption
Age 4 to 12	0.74	0–1	
Age 13 to 17	0.5	0–1	
Age 18 and over	0.375	0–1	
Medication	0.6552	0.4567–0.821	[25] ^4^

^1^ Modeled a combined function for adherence rate and treatment effect: (1-(adherence rate * treatment effect)). For medication combined with diet, the value of the function for diet treatment and medication treatment were compared and the higher value was used. ^2^ Based on Markov trace. Assumed treatment effect is consistent while adherence varies. ^3^ Individuals with PKU that are non-responsive to medication were treated with diet treatment only. ^4^ 95% confidence intervals estimated assuming a binomial distribution.

**Table 2:** Costs, 2017 US Dollars.

	Base-Case	Range for Sensitivity Analysis	Data Source
**Newborn screening and follow-up confirmatory testing**			
Screening test	4.87	1.31–14.00	[26]
Confirmatory testing	114.48	--	^1^
**Interventions**			
Diet treatment ^2^	2696–5100	--	[19]
Medication ^3^	15,142–171,713	--	[27,28,29,30]
**Laboratory and developmental testing**			
Laboratory testing, PKU			
Age 0 to 1	3870	--	[31,32] ^4^
Age 2 to 17	1290	--	
Age 18 and above	595	--	
Laboratory testing, hyperphe			
Age 0 to 1	248	--	
Age 2 to 4	198	--	
Age 5 and above	50	--	
Developmental testing	16	--	[33]; expert opinion ^5^
**Special education**			
Tutoring, mild impairment	1507	--	[34,35], assumption ^6^
Special education, age 5 to 17, moderate impairment	10,517	--	[36,37]
**Average hourly wage**	26.31	--	[34]

^1^ Personal communication with the Michigan Department of Health and Human Services (MDHHS). ^2^ Costs varied by age, includes low protein food and medical formula, see Table S2 for more detail. ^3^ Sapropterin, costs varied by age, see Table S4 for detail. ^4^ Tests include amino acids (CPT 82131), tyrosine (CPT 84510), and Phe (CPT 84030). Testing frequencies for those with PKU were age 0–1 = 78/yr; age 2–17 = 26/yr; age 18+ = 12/year. Testing frequencies for those with hyperphe were age 0–1 = 5/yr; age 2–17 = 4/yr; age 18+ = 1/year. ^5^ Yearly average for tests given every 3 years. Tests included neurobehavioral status exam (CPT 96116), neuropsychological testing (CPT 96118), and developmental testing, extended (CPT 96111). ^6^ 2 h per week of tutoring.

**Table 3:** Quality of Life Adjustments.

PKU Health State	Utility Weight		
	Base-Case	Range for Sensitivity Analysis	Data Source
Moderate/severe ^1^			
Age 0–17	0.564	0.506–0.623	[20]
Age 18+	0.679	0.628–0.730	
Mild ^1^			
Age 0–17	0.639	0.581–0.696	
Age 18+	0.808	0.762–0.852	
**Caregiver disutility** ^2^			
Moderate/severe	0.120	0.079–0.160	[20]
Mild	0.110	0.072–0.148	

^1^ Community sample ^2^ Caregiver disutility are assumed to be 0 for the health state “No/few deficits”, and 1 for health state “Dead”.

**Table 4:** Base case results (partial adherence, cohort size: 1000 individuals).

Strategies	Cost ($USD)	Incremental Cost	QALYs	Incremental QALYs	ICER ($/QALY)
CI/diet	15,332	–	30,468.921	–	–
NBS/diet	17,471	2139	30,469.255	0.334	6408
CI/diet with medication	80,865	63,394	30,468.922	–0.333	dominated
NBS/diet with medication	83,003	65,532	30,469.259	0.004	16,135,442

NBS: Newborn screening; CI: Clinical identification; QALY: Quality adjusted life year; ICER: incremental cost effectiveness ratio.

## Data Availability

The data presented in this study are available on request from the corresponding author.

## Supplementary Materials

The following are available online at https://www.mdpi.com/article/10.3390/children8050381/s1. We have included a supplementary material file along with the submission, including: Figure S1: Proportion of Hypothetical Cohort of Individuals with PKU by Health State and Age–diet treatment only (no medication), full adherence, Figure S2: Sensitivity analysis results for NBS/diet with medication when compared with NBS/diet, Table S1: Epidemiology inputs, Table S2: Costs, Table S3: Quality of life adjustments, Table S4: Medication (sapropterin) cost, Table S5: Mortality, Table S6: Impact Inventory, Table S7: One–way sensitivity analysis results (ICER: $/QALYs), Table S8: Scenario analysis, healthcare sector perspective, cohort size: 1000 individuals, Table S9: Scenario analysis, full adherence and alternative adherence, cohort size: 1000 individuals, Table S10: Scenario analysis, experienced individuals with PKU sample utility, cohort size: 1000 individuals, Table S11: Scenario analysis, Veterans Administration medication cost, cohort size: 1000 individuals, Table S12: Scenario analysis, including caregiver disutility, cohort size: 1000 individuals, Table S13: Scenario analysis, partial reverse IQ for late treated individuals with PKU, cohort size: 1000 individuals.
