# Supplementary material for: Newborn Screening and Treatment of Phenylketonuria: Projected Health Outcomes and Cost-Effectiveness
Source: Children (Basel). 2021 May 12;8(5):381. doi: 10.3390/children8050381 (PMC8151371; doi:10.3390/children8050381)
Supplement: Supplementary file 1 [file children-08-00381-s001.zip › children-1187000-supplementary.pdf]

Supplementary Materials

Newborn Screening and Treatment of Phenylketonuria: Projected Health Outcomes and Cost-Effectiveness

a. Clinical identification[1,2] b. Newborn screening[2]

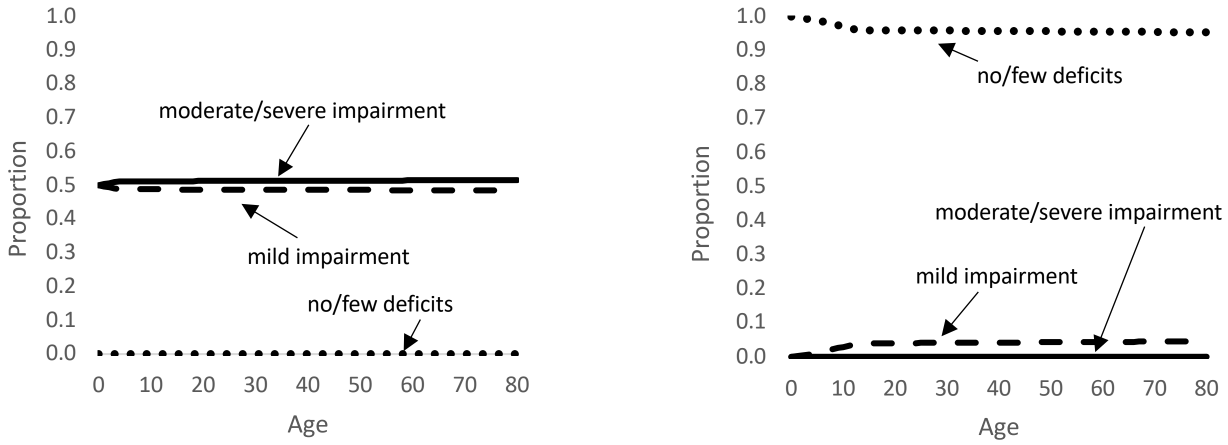

Figure S1. Proportion of Hypothetical Cohort of Individuals with PKU by Health State and Age - diet treatment only (no medication), full adherence

[1] Individuals with PKU that are identified through clinical identification start with mild or moderate/severe impairment. [2] These figures only reflect the proportion of individuals alive at that age and does not include those that have died.

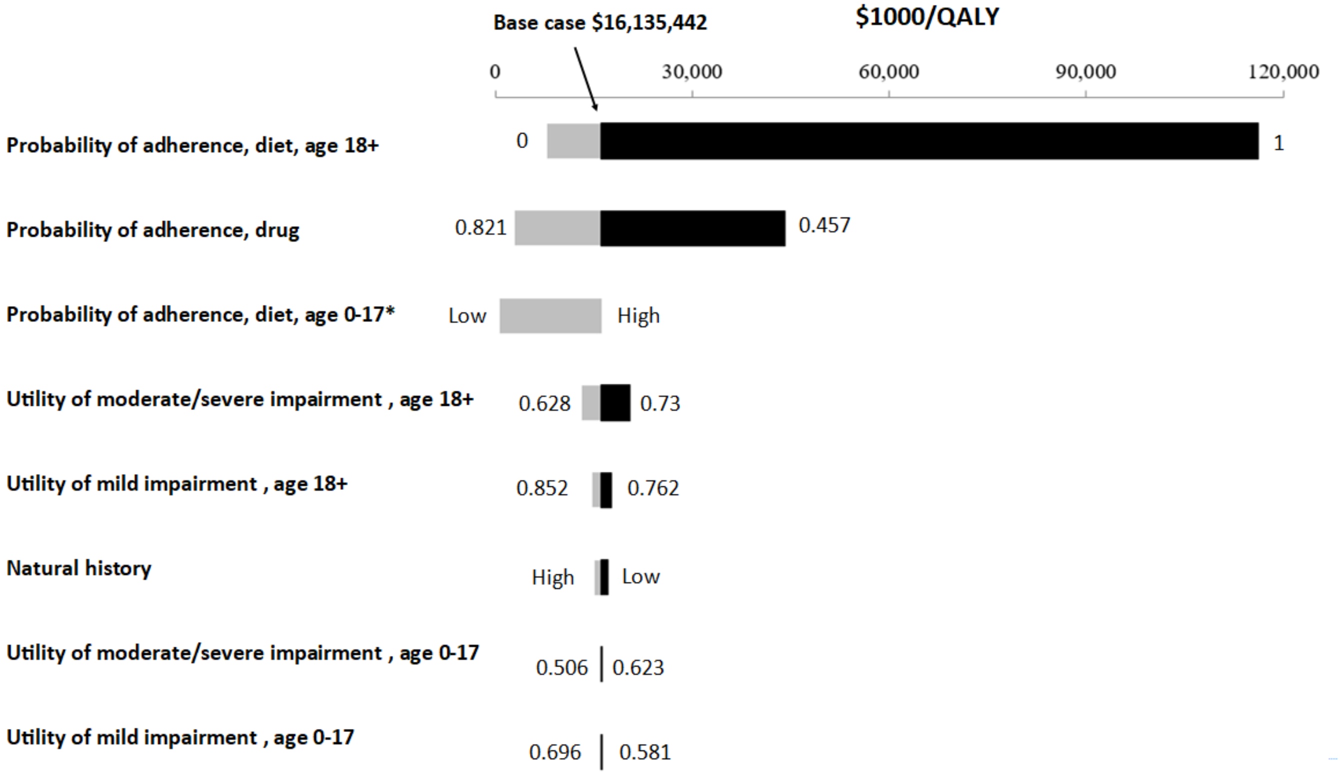

Figure S2. Sensitivity analysis results for NBS/diet with medication when compared with NBS/diet

*When using all upper bound values, the ICER is $13,592,983/QALYs, with cost of $65,534 and 0.0048 QALYs per 1000 newborns.

Table S1 Epidemiology inputs

	Base-case	Range for sensitivity analysis	Data source
**Newborn screening**			
Probability false negative screen	0	--	[1, 2]
Probability positive screen	0.0002064	0.0001308- 0.0003097	[3]; range: 95% confidence intervals estimated assuming a binomial distribution
Probability positive screen, confirmatory testing| positive initial screen	0.4782609	0.2681962 - 0.694122	[3]; range: 95% confidence intervals estimated assuming a binomial distribution
Probability PKU| positive confirmatory test	0.5454545	0.2337936 - 0.8325119	[3]; range: 95% confidence intervals estimated assuming a binomial distribution
**Probability phe level 360-600|PKU**	0.3770197	0.3366195 - 0.418732	[4]; range: 95% confidence intervals estimated assuming a binomial distribution
**Transition probabilities, natural history**			
No deficits to mild deficits			
Age 0 to 3	0.2	0.18 - 0.22	Estimated from Smith et al.,1974 [5] and Koch et al.,1999 [6] and modified based on expert opinion[1]; range calculated as plus/minus 10%
Age 4 to 12	0.36	0.324 - 0.396	
Age 13 and above	0.01	0.009 - 0.011	
Mild deficits to moderate deficits			
Age 0 to 3	0.59	0.531 - 0.649	
Age 4 and above	0.0098	0.00882 - 0.01078	
Moderate deficits to moderate deficits	1	--	
**Treatment**[2]			
Probability of responding to medication | phe level 360-600, NBS	0.81	0.79 - 0.83	[4]; range: [4]
Probability of responding to medication | phe level > 600, NBS/CI	0.315	0.07 - 0.6	[4]; range: [4]
Treatment effect – Diet treatment	0.99	--	Assumed based on Markov trace; Assume Treatment effect is consistent, and adherence is what variates
Treatment effect - Medication	1	--	Assumed; Individuals with PKU with no medication effect are already ruled out by the probability of unresponsive to medication treatment (treated with diet treatment only)
**Adherence rate**[2]			
Diet treatment			
Age 0 to 3	0.88	0 - 1	
Age 4 to 12	0.74	0 - 1	[7]; range: assumed
Age 13 to 17	0.5	0 - 1	
Age 18 and over	0.375	0 - 1	
Medication	0.6552	0.4567 - 0.8201	[8]; range: 95% confidence intervals estimated assuming a binomial distribution

[1] From Smith et al.,1974 [5]: If untreated, individuals with PKU reach IQ<85/Stage III/moderate/severe by age 3; From Koch et al.,1999 [6]: probability of transferring from Stage II to Stage III: 0.0098459 (1 year probability) (0.13793 -15 year probability); Expert opinion: (1) For untreated (clinical identification): have at least 1% in mild, (2) For early treated: have 50%,50% in no/few deficit and mild respectively by age 10; All values were adjusted to incorporate mortality rate from life table [9].

[2] In the model, a function for adherence rate and treatment effect combined was set up in model: (1 - (adherence rate * treatment effect)). For diet treatment combined with medication, the value of the function for diet treatment and medication were compared and the higher value was used.

Table S2 Costs

	Base-case	Range for sensitivity analysis	Data source
**Newborn screening and follow-up confirmatory testing**			
Screening test	4.87	1.31 - 14.00	[10]
Confirmatory testing	114.48	--	[1]
**Interventions**			
Diet treatment[2]	2,696 - 5,100	--	[11]
Medication[3]	15,142 - 171,713	--	[12-15]
**Laboratory and developmental testing**			
Laboratory testing, PKU			
Age 0 to 1	3870.36	--	Cost:[16]; frequency: [17][4]
Age 2 to 17	1290.12	--	
Age 18 and above	595.44	--	
Laboratory testing, hyperphe			
Age 0 to 1	248.10	--	
Age 2 to 4	198.48	--	
Age 5 and above	49.62	--	
Developmental testing	161.40	--	Cost: [18]; frequency: expert opinion[5]
**Provider visits**			
Dietician			
Age 0 to 17	88.47	0 - 227.50	[11, 18][6]
Age 18 and above	499.24	6.32 - 1390.29	
Geneticist			
Age 0 to 17	13.22	0 - 26.45	[11, 19] ; expert opinion [7]
Age 18 and above	19.84	6.62 - 33.06	
Metabolic specialists, PKU			
Age 0 to 17	194.34	168.43 - 207.30	[11, 18][8]
Age 18 and above	194.34	142.52 - 259.12	
Metabolic specialists, hyperphe			[18], expert opinion[8]
Age 0 to 2	129.56	--	
Age 3 and above	64.78	--	
Primary care provider, PKU			[11, 18][9]
Age 0 to 17	69.09	37.69 - 100.49	
Age 18 and above	113.05	75.37 - 150.73	
Primary care provider, hyperphe			[18], expert opinion[9]
Age 0 to 2	62.81	--	
Age 3 and above	31.40	--	

**Visit time**			
Dietician			
Age 0 to 17	0.1	0 - 0.2	[11]
Age 18 and above	2.8	1.5 - 5	
Geneticist			
Age 0 to 17	0.3	0 - 0.9	
Age 18 and above	0.8	0.4 - 1.7	
Metabolic specialists, PKU			
Age 0 to 17	5.9	4.6 - 6.9	
Age 18 and above	6	4.7 - 7.3	
Metabolic specialists, hyperphe			
Age 0 to 2	5.9	--	
Age 3 and above	6.0	--	
Primary care provider, PKU			
Age 0 to 17	1.3	1.1 - 1.6	
Age 18 and above	1.2	1 - 1.4	
Primary care provider, hyperphe			
Age 0 to 2	1.3	--	
Age 3 and above	1.2	--	
**Food and food preparation time cost**			
Low protein food, out of pocket			
Age 0 to 17	1,166	703 - 1,695	[11]
Age 18 and above	386	128 - 705	
Low protein food, retail			
Age 0 to 17	1,238	730 - 1,792	
Age 18 and above	151	20 - 358	
Medical formula[10], out of pocket			
Age 0 to 17	0	--	
Age 18 and above	114	0 - 344	
Medical formula[10], retail			
Age 0 to 17	2,696	1,750 - 3,680	
Age 18 and above	4,205	2,896 - 5,603	
Time cost, food preparation			
Age 0 to 17	5,709	4,085 - 7,472	
Age 18 and above	6,500	4,079 - 9,507	
**Special education cost**			
Tutoring, mild impairment	1,507	--	[20, 21][11]
Special education, age 5 to 17, moderate impairment	10,517	--	[22, 23]
**Average hourly wage**	26.31	--	[20]

[1] Personal communication with the Michigan Department of Health and Human Services (MDHHS)

[2] Depending on age, includes low protein food and medical formula, see Food and food preparation time cost item below for more detail.

[3] Sapropterin, depending on age, see Table S1.3 for detail.

[4] Tests include amino acids (CPT 82131), tyrosine (CPT 84510), and Phe (CPT 84030). Testing frequencies for those with PKU were: age 0-1= 78/yr; age 2-17= 26/yr; age 18+=12/year. Testing frequencies for those with hyperphe were: age 0-1= 5/yr; age 2-17= 4/yr; age 18+=1/year.

[5] Annual average assuming tests are conducted every 3 years. Tests included neurobehavioral status exam (CPT 96116), neuropsychological testing (CPT 96118), and developmental testing, extended (CPT 96111)

[6] CPT 97802 and 97803. Frequency of visits was 1.4/yr for children and 7.9/yr for adults.
[7] CPT 96040. Frequency of visits was 0.2/yr for children and 0.3/yr for adults.
[8] CPT 99213. Frequency of visits for those with PKU was 1.5/yr for children and adults. Frequency of visits for those with hyperphe was 1/yr for children 0-2 yr and 0.5/yr for individuals 3+ yr

[9] CPT 99215. Frequency of visits for those with PKU was 1.1/yr for children and 1.8/yr for adults. Frequency of visits for those with hyperphe was 1/yr for children 0-2 yr and 0.5/yr for individuals 3+ yr

[10] Includes amino acid mixture supplementation.
[11] 2 hours per week of tutoring

Table S3 Quality of life adjustments

PKU Health State	Utility Weight		
	**Base-case**	**Range for sensitivity analysis**	**Data source**
**Community sample**			
Moderate/severe			
Age 0 -17	0.564	0.506 - 0.623	
Age 18+	0.679	0.628 - 0.730	
Mild			[24]
Age 0-17	0.639	0.581 - 0.696	
Age 18+	0.808	0.762 - 0.852	
**Experienced PKU sample**			
Moderate/severe			
Age 0 -17	0.569	0.39 - 0.739	
Age 18+	0.812	0.737 - 0.878	
Mild			[24]
Age 0-17	0.622	0.441 - 0.792	
Age 18+	0.916	0.857 - 0.965	
**Caregiver disutility[1]**			
Moderate/severe	0.120	0.079-0.160	[24]
Mild	0.110	0.072-0.148	

[1] Caregiver disutility are assumed to be 0 for the health state “No/few deficits”, and 1 for health state “Dead”.

Table S4 Medication (sapropterin) cost

Age	Average wholesale price	Veterans Administration Price
0	15,142	12,246
1	22,637	18,307
2	27,939	22,596
3	32,528	26,307
4	36,912	29,853
5	43,132	34,883
6	48,842	39,501
7	54,553	44,119
8	64,444	52,119
9	73,519	59,458
10	82,696	66,880
11	95,951	77,601
12	105,842	85,600
13	118,282	95,661
14	127,969	103,495
15	136,025	110,010
16	140,205	113,391
17	143,876	116,360
18	149,586	120,978
19	150,402	121,638
20	159,885	129,307
30	170,490	137,883
40	170,490	137,883
50	171,713	138,873
60	171,204	138,461
70	164,474	133,018
80	147,037	118,916

Calculated based on Medication (sapropterin) cost per 100 mg tab, recommended initial dose and maintenance dose per kg from literature, and weight (kg) from the Anthropometric Reference Data for Children and Adults: United States 2007-2010. [12-15]

Table S5 Mortality [9]

Age	Mortality	Age	Mortality	Age	Mortality	Age	Mortality	Age	Mortality
0	0.005958	21	0.000793	42	0.001971	63	0.010905	84	0.074866
1	0.000422	22	0.000856	43	0.002144	64	0.011695	85	0.083543
2	0.000255	23	0.000894	44	0.002348	65	0.012556	86	0.093322
3	0.000186	24	0.000913	45	0.002570	66	0.013508	87	0.104041
4	0.000159	25	0.000928	46	0.002815	67	0.014581	88	0.115743
5	0.000145	26	0.000947	47	0.003103	68	0.015815	89	0.128461
6	0.000129	27	0.000970	48	0.003431	69	0.017230	90	0.142215
7	0.000116	28	0.001000	49	0.003782	70	0.018838	91	0.157012
8	0.000104	29	0.001035	50	0.004138	71	0.020667	92	0.172842
9	0.000095	30	0.001073	51	0.004496	72	0.022677	93	0.189673
10	0.000091	31	0.001112	52	0.004873	73	0.024820	94	0.207455
11	0.000098	32	0.001149	53	0.005281	74	0.027179	95	0.226114
12	0.000122	33	0.001184	54	0.005723	75	0.029681	96	0.245553
13	0.000166	34	0.001221	55	0.006200	76	0.032686	97	0.265656
14	0.000227	35	0.001269	56	0.006695	77	0.036220	98	0.286286
15	0.000292	36	0.001331	57	0.007203	78	0.040148	99	0.307291
16	0.000359	37	0.001407	58	0.007722	79	0.044507	100	1.000000
17	0.000437	38	0.001494	59	0.008260	80	0.049302		
18	0.000525	39	0.001591	60	0.008842	81	0.054486		
19	0.000616	40	0.001700	61	0.009481	82	0.060393		
20	0.000709	41	0.001826	62	0.010169	83	0.067070		

Table S6. Impact Inventory

Sector	*Type of Impact (list category within each sector with unit of measure if relevant*)*	*Included in This Reference Case Analysis from … perspective?*		*Notes on Sources of Evidence*
		Healthcare Sector	*Societal*	
Formal Healthcare sector				
Health	*Health outcomes (effects)*			
	Longevity effects	☐	☐	
	Health-related quality-of-life effects	☑	☑	Utility Weight
	Other health effects (e.g., adverse events and secondary transmissions of infections)	☐	☐	
	Medical costs			
	Paid for by third-party payers	☑	☑	Newborn screening and follow-up cost; Lab and developmental testing cost; Visit costs; Medical formula cost; Low protein food cost
	Paid for by patients Out-of-pocket	☑	☑	Medical formula cost; Low protein food cost
	Future related medical costs (payers and patients)	☑	☑	Lab and development cost; Visit costs; Medical formula cost; Low protein food
	Future unrelated medical costs (payers and patients)	☐	☐	
Informal Healthcare sector				
Health	Patient time costs	NA	☑	Visit time cost (Wage cost)
	Unpaid caregiver time costs	NA	☑	Visit time cost (Wage cost); Time cost of food preparation
	Transportation costs	NA	☐	
Non-healthcare sectors (with examples of possible items)				

Productivity	Labor market earnings lost	NA	☐	Utility weights included productivity loss
	Cost of unpaid lost productivity due to illness	NA	☐	
	Cost of uncompensated household production	NA	☐	
Consumption	Further consumption unrelated to health	NA	☐	
Social services	Cost of social services as part of intervention	NA	☐	Not included in the analysis but could be included for moderate/severe impairment
Legal/criminal justice	Number of crimes related to intervention	NA	☐	Not relevant to PKU
	Cost of crimes related to intervention	NA	☐	
Education	Impact of intervention on educational achievement of population	NA	☑	Special education cost
Housing	Cost of intervention on home improvements (e.g., removing lead paint)	NA	☐	Not relevant to PKU
Environment	Production of toxic waste or pollution by intervention	NA	☐	Not relevant to PKU
Other (specify)	Other impacts	NA	☐	
*Categories listed are intended as examples for analysts.<br>Abbreviation: NA = Not applicable				

Table S7. One-way sensitivity analysis results (ICER: $/QALYs)

	Low	High	Range
**(1) NSB/diet (Base case: $6,408/QALYs)**			
Probability of positive NBS[1]	Cost saving	$38,028	Larger than $38,028
Cost of NBS[2]	Cost saving	$33,758	Larger than $33,758
Probability of true positive NBS[3]	Cost saving	$33,534	Larger than $33,534
Probability of having PKU when positive NBS	$267	$30,147	$29,880
Probability of adherence, diet, age 0 to 17	$4,584	$17,064	$12,480
Utility of moderate/severe impairment, age 0 to 17	$5,935	$6,972	$1,036
Utility of moderate/severe impairment, age 18+	$5,941	$6,953	$1,012
Utility of mild impairment, age 18+	$6,211	$6,627	$416
Natural History	$6,214	$6,600	$386
Probability of adherence, diet, age 18+	$6,199	$6,527	$329
**(2) CI/diet with medication (Base case: dominated)**			
Probability of adherence, diet, age 0 to 17	dominated	dominated	$297,621
Probability of positive NBS	dominated	dominated	$194,901
Probability of true positive NBS	dominated	dominated	$174,595
Cost of NBS[4]	dominated	dominated	$158,854
Probability of responding to medication, Phe level over 600	dominated	dominated	$130,111
Probability of adherence, drug	dominated	dominated	$110,452
Utility of moderate/severe impairment, age 0 to 17	dominated	dominated	$30,922
Probability of having PKU when positive NBS	dominated	dominated	$29,985
Utility of moderate/severe impairment, age 18+	dominated	dominated	$29,661
Probability of Phe level 360-600 when confirmed PKU	dominated	dominated	$16,014
**(3) NSB/diet with medication (Base case: $16,135,442/QALYs)**			
Probability of adherence, diet, age 18+	$7,870,283	$116,199,632	$108,329,349
Probability of adherence, drug	$2,998,427	$44,122,066	$41,123,638

Probability of adherence, diet, age 0 to 17[5]	$773,931	$13,592,983	$12,819,052
Utility of moderate/severe impairment, age 18+	$13,263,829	$20,594,043	$7,330,213
Utility of mild impairment, age 18+	$14,861,066	$17,724,449	$2,863,383
Natural History	$15,219,198	$17,270,717	$2,051,518
Utility of moderate/severe impairment, age 0 to 17	$16,098,325	$16,173,376	$75,051
Utility of mild impairment, age 0 to 17	$16,120,648	$16,150,524	$29,876

[1] NSB/diet was considered cost-saving at the low value assumed in the sensitivity analysis. CI/diet was dominated due to higher costs and lower QALYs. (Value range of variable probability of positive NBS: 0.0001308 to 0.0003097)

[2] NSB/diet was considered cost-saving at the low value assumed in the sensitivity analysis. CI/diet was dominated due to higher costs and lower QALYs. (Value range of cost of NBS: 1.305132216 to 14.00179888)

[3] NSB/diet was considered cost-saving at the low value assumed in the sensitivity analysis. CI/diet was dominated due to higher costs and lower QALYs. (Value range of true positive NBS: 0.2681962 to 0.694122)

[4] When at high value, the ICER was larger than the base-case value but still remain dominated.

[5] When using all high value, the ICER is $13,592,983/QALYs, with cost of $65,534 and 0.0048 QALYs per 1000 newborns.

NBS: Newborn screening; CI: Clinical identification; QALY: Quality adjusted life year; ICER: incremental cost effectiveness rate

Table S8. Scenario analysis, healthcare sector perspective, cohort size: 1,000 individuals

**Strategies**	**Cost ($USD)**	**Incremental cost**	**QALYs**	**Incremental QALYs**	**ICER ($/QALY)**
(1) Excluding low protein food cost					
CI/diet	4,631	-	30,468.921	-	-
NBS/diet	9,752	5,121	30,469.255	0.334	15,339
CI/diet with medication	70,164	60,412	30,468.922	-0.333	dominated
NBS/diet with medication	75,286	65,534	30,469.259	0.004	16,135,836
(2) Including low protein food cost					
CI/diet	5,359	-	30,468.921	-	-
NBS/diet	10,481	5,121	30,469.255	0.334	15,339
CI/diet with medication	70,893	60,412	30,468.922	-0.333	dominated
NBS/diet with medication	76,014	65,534	30,469.259	0.004	16,135,836

NBS: Newborn screening; CI: Clinical identification; QALY: Quality adjusted life year; ICER: incremental cost effectiveness ratio

Table S9. Scenario analysis, full adherence and alternative adherence, cohort size: 1,000 individuals

Strategies	Cost ($USD)	Incremental cost	QALYs	Incremental QALYs	ICER ($/QALY)
(1) Full adherence rate					
CI/diet	24,755	-	30,468.948	-	-
NBS/diet	27,060	2,305	30,469.466	0.518	4,452
CI/diet with medication	124,753	97,693	30,468.949	-0.517	dominated
NBS/diet with medication	127,074	100,014	30,469.472	0.006	15,559,374
(2) Alternative adherence rate for age group 18 and above					
Adherence rate = 0.5					
CI/diet	16,791	-	30,468.922	-	-
NBS/diet	18,931	2,139	30,469.258	0.336	6,367
CI/medication with diet	82,324	63,394	30,468.923	-0.335	dominated
NBS/medication with diet	84,463	65,532	30,469.261	0.003	25,647,548
Adherence rate = 0.74					
CI/diet	19,594	-	30,468.924	-	-
NBS/diet	21,733	2,139	30,469.264	0.340	-
CI/medication with diet	85,127	63,394	30,468.924	-0.340	6,287
NBS/medication with diet	87,265	65,532	30,469.265	0.001	dominated
Adherence rate = 0.88					
CI/diet	21,228	-	30,468.925	-	-
NBS/diet	23,368	2,139	30,469.268	0.343	6,240
CI/medication with diet	86,761	63,394	30,468.925	-0.343	dominated
NBS/medication with diet	88,900	65,532	30,469.268	0.001	118,072,514
Adherence rate = 1					
CI/diet	22,630	-	30,468.926	-	-
NBS/diet	24,769	2,139	30,469.271	0.345	6,199
CI/medication with diet	88,162	63,394	30,468.926	-0.345	dominated
NBS/medication with diet	90,301	65,532	30,469.271	0.001	116,199,632

NBS: Newborn screening; CI: Clinical identification; QALY: Quality adjusted life year; ICER: incremental cost effectiveness ratio

Table S10. Scenario analysis, experienced individuals with PKU sample utility, cohort size: 1,000 individuals

Strategies	Cost ($USD)	Incremental cost	QALYs	Incremental QALYs	ICER ($/QALY)
CI/diet	15,332	-	30,469.029	-	-
NBS/diet	17,471	2,139	30,469.319	0.290	7,380
CI/diet with medication	80,865	63,394	30,469.030	-0.289	dominated
NBS/diet with medication	83,003	65,532	30,469.322	0.003	25,000,119

NBS: Newborn screening; CI: Clinical identification; QALY: Quality adjusted life year; ICER: incremental cost effectiveness ratio

Table S11. Scenario analysis, Veterans Administration medication cost, cohort size: 1,000 individuals

Strategies	Cost ($USD)	Incremental cost	QALYs	Incremental QALYs	ICER ($/QALY)
CI/diet	15,332	-	30,468.921	-	-
NBS/diet	17,471	2,139	30,469.255	0.334	6,408
CI/diet with medication	68,331	50,860	30,468.922	-0.333	dominated
NBS/diet with medication	70,470	52,999	30,469.259	0.004	13,049,437

NBS: Newborn screening; CI: Clinical identification; QALY: Quality adjusted life year; ICER: incremental cost effectiveness rate

Table S12. Scenario analysis, including caregiver disutility, cohort size: 1,000 individuals

Strategies	Cost ($USD)	Incremental cost	QALYs	Incremental QALYs	ICER ($/QALY)
CI/diet	15,332	0	30,470.370	-	-
NBS/diet	17,471	2,139	30,470.799	0.429	4,990
CI/diet with medication	80,865	63,394	30,470.372	-0.428	dominated
NBS/diet with medication	83,003	65,532	30,470.804	0.005	12,430,733

NBS: Newborn screening; CI: Clinical identification; QALY: Quality adjusted life year; ICER: incremental cost effectiveness rate

When caregiver disutility was 0.079 and 0.072 for moderate/severe and mild impairment respectively, the ICER for NBS/diet was $5,397/QALY, and for NBS/diet with medication $13,479,669/QALY, while CI/diet with medication remain dominated. When caregiver disutility was 0.160 and 0.148 for moderate/severe and mild impairment respectively, the ICER for NBS/diet was $4,650/QALY and for NBS/diet with medication $11,568,691/QALY, while CI/diet with medication remain dominated.

Table S13. Scenario analysis, partial reverse IQ for late treated individuals with PKU, cohort size: 1,000 individuals

Strategies	Cost ($USD)	Incremental cost	QALYs	Incremental QALYs	ICER ($/QALY)
CI/diet	13,819	-	30,469.020	-	-
NBS/diet	17,471	3,652	30469.255	0.2355	15,512

NBS: Newborn screening; CI: Clinical identification; QALY: Quality adjusted life year; ICER: incremental cost effectiveness rate

Analysis was done by assuming late treated individuals with PKU had a probability of 0.542 in transferring to a better health state when they were 1 year old (when the symptoms appeared and they were diagnosed and immediately received diet treatment), this assumption was based on data from Koch et al and expert opinion.[6]

References

1. Pandor, A., et al., *Clinical effectiveness and cost-effectiveness of neonatal screening for inborn errors of metabolism using tandem mass spectrometry: a systematic review.* Health Technol Assess, 2004. **8**(12): p. iii, 1-121.
2. Chace, D.H., et al., *Use of phenylalanine-to-tyrosine ratio determined by tandem mass spectrometry to improve newborn screening for phenylketonuria of early discharge specimens collected in the first 24 hours.* Clin Chem, 1998. **44**(12): p. 2405-9.
3. Michigan Department of Health and Human Services MDHHS, *Michigan Newborn Screening Program Annual Report 2015*. 2017.
4. Fiege, B. and N. Blau, *Assessment of tetrahydrobiopterin (BH4) responsiveness in phenylketonuria.* J Pediatr, 2007. **150**(6): p. 627-30.
5. Smith, I. and O.H. Wolff, *Natural history of phenylketonuria and influence of early treatment.* Lancet, 1974. **2**(7880): p. 540-4.
6. Koch, R., et al., *Long-term beneficial effects of the phenylalanine-restricted diet in late-diagnosed individuals with phenylketonuria.* Mol Genet Metab, 1999. **67**(2): p. 148-55.
7. Jurecki, E.R., et al., *Adherence to clinic recommendations among patients with phenylketonuria in the United States.* Mol Genet Metab, 2017. **120**(3): p. 190-197.
8. Rohr, F., et al., *Adherence to tetrahydrobiopterin therapy in patients with phenylketonuria.* Mol Genet Metab, 2015. **114**(1): p. 25-8.
9. Arias, E., M. Heron, and J. Xu, *United States Life Tables, 2013.* Natl Vital Stat Rep, 2017. **66**(3): p. 1-64.
10. PricewaterhouseCoppers L.L.P. (P.w.C.). *Newborn Screening Programs: An Overview of Costs and Financing*. 2002; Available from: https://www.marchofdimes.org/chapterassets/files/final_pwc_nbs_report2.pdf.
11. Rose, A.M., et al., *The financial and time burden associated with phenylketonuria treatment in the United States.* Molecular Genetics and Metabolism Reports, 2019. **21**: p. 100523.
12. Truven Health Analytics, *Micromedex Solutions RED BOOK*. 2016.
13. Veterans Administration (V.A.). *Pharmaceutical Prices*. 2017 [cited 2017; Available from: https://www.va.gov/opal/nac/fss/pharmPrices.asp.
14. Lee, P., et al., *Safety and efficacy of 22 weeks of treatment with sapropterin dihydrochloride in patients with phenylketonuria.* Am J Med Genet A, 2008. **146a**(22): p. 2851-9.
15. Vernon, H.J., et al., *Introduction of sapropterin dihydrochloride as standard of care in patients with phenylketonuria.* Mol Genet Metab, 2010. **100**(3): p. 229-33.
16. Centers for Medicare and Medicaid Services (C.M.S.). *Clinical Laboratory Fee Schedule 2016*. 2016; Available from: https://www.cms.gov/Medicare/Medicare-Fee-for-Service-Payment/ClinicalLabFeeSched/clinlab.html.
17. National PKU Alliance (N.P.K.U.A.). *MY PKU Binder*. 2014; Available from: https://npkua.org/Education/My-PKU-Binder.
18. Centers for Medicare and Medicaid Services (C.M.S.). *Physician Fee Schedule Search and Healthcare Common Procedure Coding System (HCPCS) code*. 2017; Available from: https://www.cms.gov/apps/physician-fee-schedule/search/search-criteria.aspx.
19. Dungan, J.Y., C.; Trivedi, A.; Bamlett Sherman, T.; Shulman, L. *Reimbursement for genetic counseling and related services*. 2017 [cited 2017; Available from: http://www.ashg.org/genetics/ashg07s/f10110.htm.
20. United States Department of Labor. *Bureau of Labor Statistics*. 2017 [cited 2017; Available from: https://www.bls.gov/.
21. Sylvan learning. *Sylvan learning program*. 2017; Available from: http://www.sylvanlearning.com/tutoring/how-tutoring-works/what-to-expect/pricing.
22. National Education Association. *Background of Special Education and the Individuals with Disabilities Education Act (IDEA)*. 2004 [cited 2017; Available from: http://www.nea.org/home/19029.htm.

23. American Institutes for Rsearch (A.I.R.). *Center for Special Education Finance (CSEF)*. 2017 [cited 2017; Available from: https://www.air.org/project/center-special-education-finance-csef.
24. Simon, N.J., et al., *Health utilities and parental quality of life effects for three rare conditions tested in newborns.* J Patient Rep Outcomes, 2019. **3**(1): p. 4.
